# Loss of miR-100 and miR-125b results in cancer stem cell properties through IGF2 upregulation in hepatocellular carcinoma

**DOI:** 10.1038/s41598-020-77960-9

**Published:** 2020-12-08

**Authors:** Hyang Sook Seol, Yoshimitsu Akiyama, San-Eun Lee, Shu Shimada, Se Jin Jang

**Affiliations:** 1 Asan Institute for Life Science, Asan Medical Center, Seoul, 05505 South Korea; 2 Department of Molecular Oncology, Graduate School of Medical and Dental Sciences, Tokyo Medical and Dental University, Tokyo, 113-8519 Japan; 3 Department of Pathology, Asan Medical Center, University of Ulsan College of Medicine, 05505, Seoul, South Korea

**Keywords:** Cancer, Cancer stem cells

## Abstract

Stemness factors control microRNA expression in cancer stem cells. Downregulation of miR-100 and miR-125b is associated with tumor progression and prognosis of various cancers. Comparing miRNA profiling of patient-derived tumorsphere (TS) and adherent (2D) hepatocellular carcinoma cells, miR-100 and miR-125b are identified to have association with stemness. In TS cells, miR-100 and miR-125b were downregulated comparing to 2D cells. The finding was reproduced in Hep3B cells. Overexpression of stemness factors NANOG, OCT4 and SOX2 by introduction of gene constructs in Hep3B cells suppressed these two miRNA expression levels. Treatment of chromeceptin, an IGF signaling pathway inhibitor, decreased numbers of TS and inhibited the AKT/mTOR pathway. Stable cell line of miR-100 and miR-125b overexpression decreased IGF2 expression and inhibited tumor growth in the xenograft model. In conclusion, miR-100 and miR-125b have tumor suppressor role in hepatocellular carcinoma through inhibiting IGF2 expression and activation of the AKT/mTOR pathway.

## Introduction

Micro RNAs (miRNAs) are small non-coding RNAs that function as critical regulators of gene expression and are involved in diverse biological processes, including cellular growth, differentiation, and homeostasis^1^. Abnormal expression of miRNAs has been commonly reported in human cancers, and it seems to play a role in overexpression of oncogenes or loss of tumor suppressor genes^2,3^. In addition, several miRNAs, such as miR-134, miR-296, and miR-470, behave as mediators of pluripotency, self-renewal, and differentiation of cancer stem cells by controlling the stemness factors NANOG, OCT4, and SOX2^4–6^.

Liver cancer ranks second and third as the cause of cancer mortality globally and in Korea, respectively^7,8^. Median survival after diagnosis is reported to be 6 to 20 months. The dismal outcome is related to high recurrence and drug resistance, which are largely due to the presence of liver cancer stem cells (CSCs)^9^. CSCs differ in their miRNA expression profile especially in miRNAs that regulate the expression of genes related to self-renewal and differentiation properties of CSCs^10^. Those differently expressed miRNAs could be significant biomarkers or potential therapeutic targets.

Since CSCs are influential in intratumoral heterogeneity, relatively homogeneous conventional cell lines may not be sufficient as a model for investigations of liver CSCs and may not represent individual patients^11,12^. Alternatively, patient-derived primarily short-term cultured cancer cells (PCCs) substantially maintain tumor heterogeneity and characteristics of the tumors of patients^13^. A tumorsphere (TS) culturing method described the enrichment of patient-derived liver CSCs using PCCs^14^. We previously established method for TS culture of short-term cultured cancer cells originated from HCC patient tissues and conventional HCC cell lines. Although tumor heterogeneity composed of tumor stem cells and differentiated tumor cells is produced by tumor stem cell function, the TS cells derived from Huh7 and SNU449 HCC cells and patient-derived cells from hepatocellular carcinoma had similar mechanism in the maintenance of stemness by upregulation of complement proteins C7 and CFH, and through NANOG, OCT4, SOX2, and c-Myc expression^15^.

In this study, we conducted miRNA array analyses to discover differentially expressed miRNAs in the patient-derived short-term cultured hepatocellular carcinoma (HCC) cells that were grown in a TS culture system or two-dimensionally as adherent cells (2D). Several differentially expressed miRNAs were identified in TSs compared to those in 2D cells, and expression of miR-125b family members and miR-100 were commonly decreased in both patient- and conventional HCC-derived TS cells compared to those in the 2D culturing cells. We also identified the common direct target genes of miR-125b and miR-100 and explored the mechanism to maintain stemness properties through these two miRNAs in HCC cells.

## Results

### hsa-miR-100 and hsa-miR-125b are downregulated in HCC CSCs

**Figure 1:**
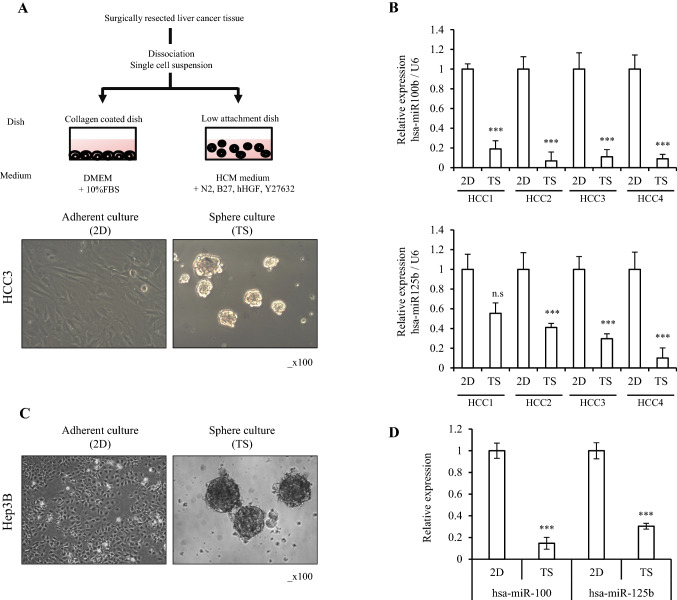
Lower expression of miR-100 and miR-125b in hepatocellular carcinoma stem cells (CSCs). (**A**) Schematic process of establishment of tumorsphere (TS) and two-dimensionally as adherent cells (2D). Hepatocellular primary cancer cells derived from patients were cultured in ultra-low attachment dishes with CSC culture medium for 7 days to enrich CSCs, as shown in the Materials and Methods. The spheres formed under the CSC culture condition, as well as monolayer cells cultured in collagen type 1 coated dishes. (**B**) Reduced expression of miR-100 and miR-125b in tumorsphere (TS) cells was quantified with specific TaqMan probe using qRT-PCR. miR-100 and miR-125b expression decreased significantly in TS cells compared with adherent cells (2D). (**C**) Hep3B formed TS under the CSC culture condition in the low attachment dish. (**D**) miR-100 and miR-125b levels were quantified with TaqMan probe using qRT-PCR in Hep3B TS cells and 2D cells. Relative expression was calculated using U6 expression as an internal control. The average (column) ± SD (bar) is indicated (^***^p < 0.001, n.s, not significant).

We first used TS and 2D cells established from four primary HCC tissues (HCC1-HCC4) reported previously in our laboratory^15^. The TS cancer cells from four HCC patients grew with a spherical shape in the low attachment dish with a specific media (Fig. 1A, right, Supplementary Fig. 1A), while spheroids were not formed in a collagen type 1 dish (Fig. 1A, left, Supplementary Fig.  1A).

We next compared the miRNA expression profiles between TS and 2D cells from the four HCC patients using the NanoString miRNA array (Table 1). The expression level of hsa-miR-125b-5p was markedly different (> 30-fold) between TS and 2D cells from the HCC2, HCC3, and HCC4 patients (Table 1). Moreover, three 2D cell lines exhibited higher hsa-miR-100-5p expression (approximately 7- to 13-fold) compared to those in TS cells. Expression of hsa-miR-125b-5p was not significantly different between TS and 2D in HCC1, but the expression of hsa-miR-100 was significantly different (3.18-fold). Consistently, the expression levels of hsa-miR-125b and hsa-miR-100 at the mature miRNA were significantly downregulated in TS cells compared to those in 2D cells in the qRT-PCR analysis (Fig. 1B). Therefore, we selected these two miRNAs for the functional analyses.

**Table 1:** Downregulated mRNA expression in tumorsphere (TS) cultured hepatocellular carcinoma cells derived from patient specimens.

HCC1		HCC2		HCC3		HCC4	
2D vs TS		2D vs TS		2D vs TS		2D vs TS	
	FC		FC		FC		FC
hsa-miR-100-5p	3.18	hsa-miR-125b-5p	124.78	hsa-miR-125b-5p	66.59	hsa-miR-125b-5p	32.59
hsa-miR-300	2.59	hsa-miR-100-5p	10.53	hsa-miR-100-5p	12.88	hsa-miR-100-5p	7.18
hsa-miR-513b	2.33	hsa-let-7b-5p	5.97	hsa-miR-125a-5p	5.37	hsa-let-7a-5p	3.13
hsa-miR-31-5p	2.27	hsa-let-7a-5p	5.11	hsa-let-7b-5p	3.96	hsa-miR-221-3p	3.11
hsa-miR-431-5p	2.14	hsa-miR-23a-3p	2.9	hsa-miR-548aj-3p	3.26	hsa-miR-409-3p	2.86
hsa-miR-212-3p	2.11	hsa-miR-4454	2.89	hsa-miR-23a-3p	2.96	hsa-let-7b-5p	2.63
hsa-miR-1263	2.05	hsa-miR-15b-5p	2.86	hsa-miR-191-5p	2.36	hsa-miR-323a-3p	2.34
		hsa-miR-1263	2.69	hsa-miR-15b-5p	2.25	hsa-miR-433	2.26
		hsa-miR-191-5p	2.32	hsa-miR-720	2.12	hsa-miR-642b-3p	2.21
						hsa-miR-491-5p	2.04
						hsa-miR-1260a	2.03
						hsa-miR-720	2.02

TS, tumor sphere; 2D, adherent cells; FC, fold-change.

We also established TS and 2D cells from Hep3B cell line under same culture condition with patient-derived TS and 2D culture. Hep3B cells also formed spheroids in the TS culturing condition but not in 2D condition (Fig. 1C). qRT-PCR analysis demonstrated that the expression levels of miR-100 and miR-125b were much lower in TS cells than in 2D cells derived from Hep3B cells (Fig. 1D). These data reconfirmed the possible association of reduced expression of miR-100 and miR-125b with the formation of spheroids when grown in the low attachment dish.

### Stemness factors regulate miR-100 and miR-125b expression by binding to the promoter region

**Figure 2:**
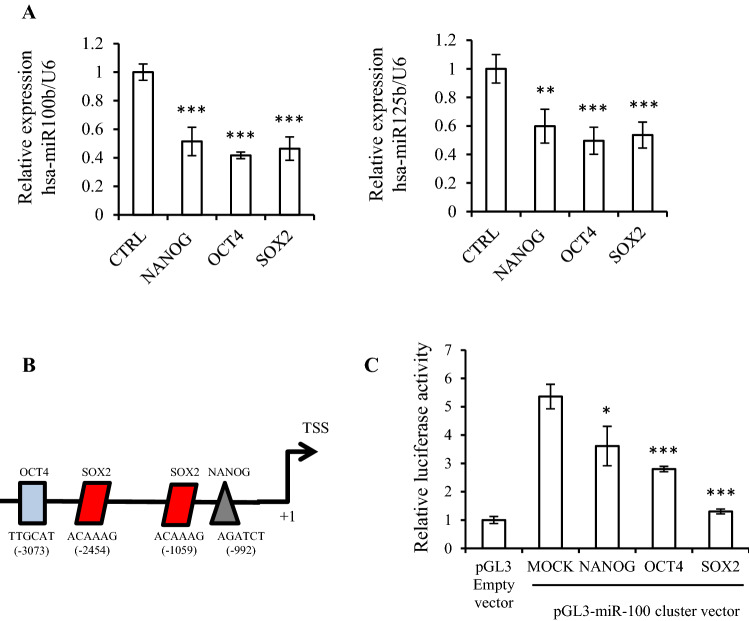
Stemness factors regulates miR-100 and -125b expression. (**A**) miR-100 and miR-125b expression levels measured after transfection of NANOG, OCT4, and SOX2 with the TaqMan probe. Relative expression was calculated using U6 expression as an internal control. (**B**) Physical map of the hsa-miR-100 promoter region for the design of chromatin immunoprecipitation (ChIP) primer. The position of the stemness binding motif designed ChIP primer. (**C**) Stemness factor transcriptional activity was assessed by pGL3-miR-100 promoter luciferase reporter in Hep3B cells. The average (column) ± SD (bar) is indicated (^*^p < 0.05, ^**^p < 0.01, ^***^p < 0.001).

To address the association of miR-100 and miR-125b expression with the expression of stemness factors, we analyzed the expression of miRNAs in Hep3B cells after transient transfection of NANOG, OCT4, or SOX2. Since one important limitation of primary cultured cancer cells is senescence after several passages of cultivation, the TS cells from Hep3B cells were used for the study of cancer stemness. The expression levels of miR-100 and miR125b were significantly decreased in Hep3B cells with overexpression of each stemness factor compared to the levels in cells transfected with empty vector (CTRL, *p* < 0.001, Fig. 2A). We previously reported that TS cells express higher levels of the NANOG, OCT4, and SOX2 stemness factor genes than 2D-cultured primary HCC cells^15^. In the present study, the expression of these three stemness markers was also increased at the mRNA level in Hep3B cells, depending on the culture periods (Supplementary Fig.  2A).

Since these two miRNAs are transcribed from miR-100HG^16^, we next constructed a pGL3-reporter vector containing approximately 3,000 bp of the upstream sequence from the transcriptional start site of miR-100HG. This region includes the putative NANOG, OCT4, and SOX2 binding sites (Fig. 2B). Transfection of the reporter vector alone (MOCK) into Hep3B cells resulted in an increase of luciferase activity compared to that of pGL3 empty vector, while the luciferase activity was attenuated when each stemness factor was overexpressed (Fig. 2C). The findings suggested that NANOG, OCT4, and SOX2 may suppress luciferase activity.

### IGF2 is overexpressed in TS cells and is regulated by stemness factors

**Figure 3:**
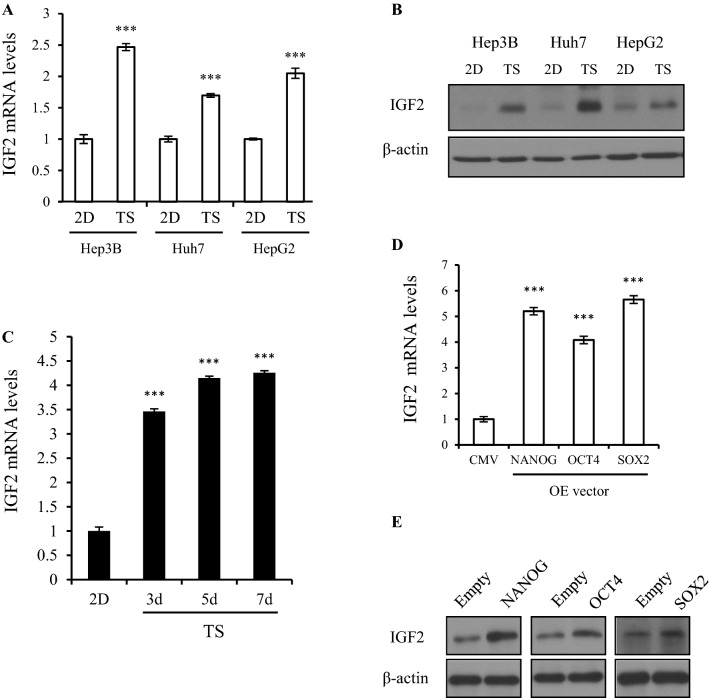
IGF2 is regulated by stemness factor. (**A**) IGF2 expression was detected by an mRNA array between tumorsphere (TS) and adherent (2D) cells. In the three liver cancer cell lines (Hep3B, HuH7, and HepG2), IGF2 mRNA (**A**) and protein (**B**) levels were measured with qRT-PCR and an immunoblot assay. Blots were cropped to show specific bands. (**C**) IGF2 expression was examined during sphere culture (3, 5, 7 days). (D-E) IGF2 mRNA (**D**) and protein (**E**) expression was detected after transient transfection of stemness factor (NANOG, OCT4, and SOX2). For qRT-PCR, relative expression was calculated using 18 s expression as an internal control. Immunoblots used β-actin as a loading control. Blotted proteins were visualized by enhanced chemiluminescence (GE Healthcare, UK) with the automatic X ray film Imaging System (Konica Minolta, Japan). Blots were cropped to show specific bands. The average (column) ± SD (bar) is indicated (^***^p < 0.001).

To verify the target candidate genes of miR-125b and miR-100, mRNA microarray analysis was performed between TS and 2D cells in Hep3B. Representative differentially expressed mRNAs are listed in Supplementary Table  1. In the TargetScan (www.targetscan.org) and microRNA (www.microRNA.com) analyses, IGF2 was detected as a common target of miR-100 and miR-125b. IGF2 is a known target of miR-100 and miR-125b in breast cancer^17,18^. To assess whether IGF2 contributes to maintaining stemness property in HCC, we examined the expression of IGF2 in TS and 2D cells from three immortalized HCC cell lines—Hep3B, HuH7, and HepG2—at the mRNA and protein levels. IGF2 expression was consistently elevated in TS compared to that in 2D (Fig. 3A,B). IGF2 mRNA levels were increased in Hep3B cells depending on the sphere culture periods (Fig. 3C). Transient overexpression of NANOG, OCT4, and SOX2 in Hep3B cells resulted in an increase of IGF2 mRNA and protein levels (Fig. 3D,E).

### IGF2 is a direct target of the miR-100/-125b cluster

**Figure 4:**
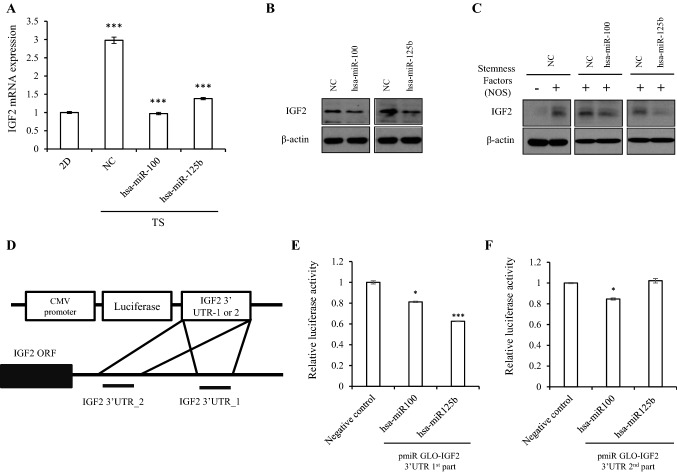
IGF2 is the target gene of miR-100 and miR-125b. (**A** & **B**) IGF2 mRNA levels (**A**) and protein levels (**B**) confirmed by qRT-PCR and immunoblots after treatment of miR-100 and -125b mimic. (**C**) Hep3B cells co-transfected with miR-100, -125b mimic (50 nM), and the stemness factor (NANOG, OCT4, and SOX2) for 48 h. Blots were cropped to show specific bands. (D) Schematic description of the miR-100 and -125b binding sites within IGF2 3′UTR. (E–F) Luciferase activity of Hep3B cells co-transfected with miR-100 or -125b mimic and negative control or plasmids containing IGF2 3′UTR 1st region (**E**) or 2nd regions (**F**). For qRT-PCR, relative expression was calculated using 18 s expression as an internal control. Immunoblots used β-actin as a loading control. The average (column) ± SD (bar) is indicated (^*^p < 0.05, ^***^p < 0.001).

We next examined the potential connection between IGF2 and miR-100/-125b in TS cells. After transfection of hsa-miR-100 and hsa-miR-125b mimics (Supplementary Fig.  3A), IGF2 expression was decreased in Hep3B-derived TS compared with the expression in the scramble negative control (NC, Fig. 4A,B). Overexpression of three stemness factor together enhanced IGF2 expression in Hep3B-derived 2D cells, and transfection of miR-100 or -125b mimic together with the NANOG, OCT4 and SOX2 stemness factors into Hep3B-derived 2D cells suppressed IGF2 expression compared to the levels using negative small interfering RNA (Fig. 4C). Since each overexpression of three stemness factors showed downregulation of miR-100 and miR-125b (Fig. 2A) and upregulation of IGF2 (Fig. 3D) in Hep3B-derived 2D cells, our data further support that stemness factors can enhance the IGF2 expression through downregulation of miR-100 and miR-125b.

The 3′UTR region of IGF2 reportedly contains miR-100 and miR-125b binding sites^19,20^. To provide direct evidence verifying that miR-100 and miR-125b downregulate IGF2 expression, we constructed two pmiR GLO vectors containing the 3′ UTR of IGF2. The 3′ UTR 1st part contained the miR-125b binding site (start 629). The 3′ UTR 2nd part contained the miR-100 binding site (Fig. 4D). The 3′UTR binding activity of both was determined in Hep3B cells. Treatment of miR-125b and miR-100 mimics attenuated IGF2 binding activity in the pmiR GLO-IGF2 with 3′ UTR 1st part compared to NC-transfected cells (Fig. 4E). For the pmiR GLO-IGF2 3′UTR 2nd part, the transfection of miR-100 led to decreased reporter activity (Fig. 4F), while miR-125b mimic transfection did not. Thus, miR-100 and miR-125b bound to the IGF2 UTR in Hep3B cells, resulting in decreased IGF2 expression.

### IGF2 regulates phosphorylation of AKT and mTOR in CSCs

**Figure 5:**
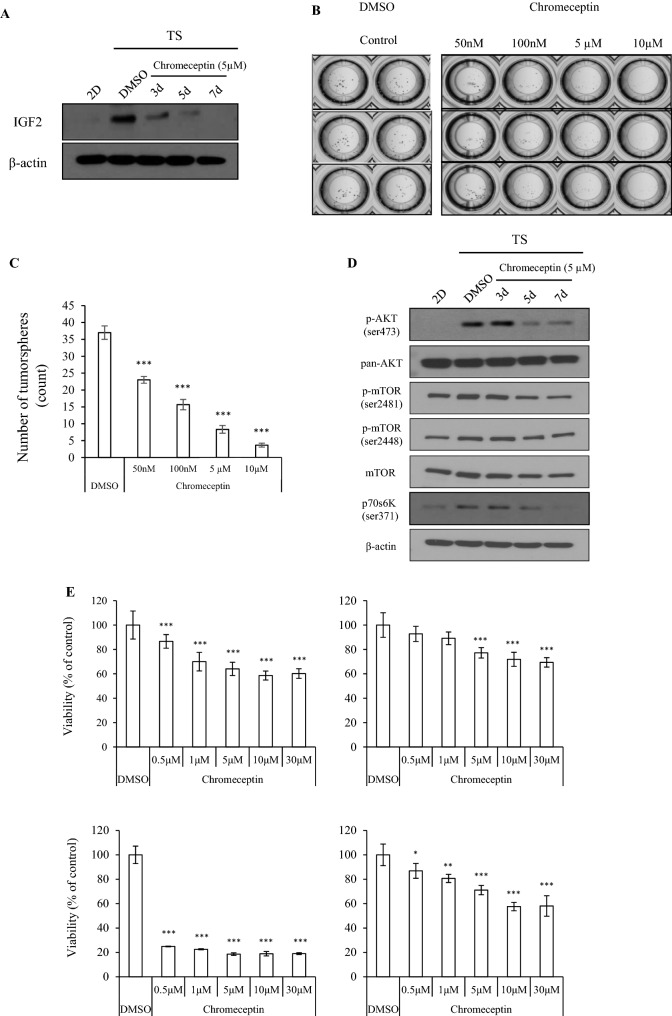
Spheres lose their stemness character with inhibition of IGF2. (**A**) Hep3B sphere forming ability was detected after treatment with different concentrations of chromeceptin. Dimethylsulfoxide (DMSO) was used as the control chemical. Blots were cropped to show specific bands. (**B**) Spheres were enumerated by microscopy examination and the data were graphed. (**C**) Graph showing tumorsphere (TS) inhibition ability after chromeceptin treatment. (**D**) Representative immunoblot of the time-dependent expression of *p*-AKT, pan-AKT, *p*-mTOR (ser2481 and ser2448), mTOR, and *p*70s6k (ser371) in 2D and TS upon treatment with chromeceptin. β-actin was used as a loading control. Blots were cropped to show specific bands. (**E**) Chromeceptin anti-cancer effect was detected with 3D cell titer GLO in hepatocellular primary cells derived patients for 72 h. The inhibition effects were normalized with DMSO treatment. Immunoblots used β-actin as a loading control. The average (column) ± SD (bar) is indicated (^*^p < 0.05, ^**^p < 0.01, ^***^p < 0.001).

To assess whether IGF2 is required for the maintenance of CSC property, Hep3B cells were treated with chromeceptin (also known as 94G6) that is a synthetic small benzochromene derivative and inhibits the IGF signaling pathway in cancer including HCC^21^. Chromeceptin/94G6 treatment suppresses IGF2 at mRNA and protein levels in hepatocyte and HCC cells^22,23^. IGF2 protein expression was undetectable in Hep3B-derived 2D cells but was markedly elevated in Hep3B-TS cells. Chromeceptin inhibited IGF2 expression in Hep3B-derived TS cells in a time-dependent manner (Fig. 5A). In addition, the number of spheres and sphere cell viability were decreased in TS cells after chromeceptin treatment in a dose-dependent manner (Fig. 5B,C). Phosphorylation levels of downstream signaling molecules protein kinase B (AKT, Ser463) and mammalian target of rapamycin (mTOR, Ser 2481 and Ser 2448) were decreased in TS cells by chromeceptin treatment (Fig. 5D). Chromeceptin also repressed the phosphorylation at ser371 in mTOR effectors ribosomal protein S6 kinase (p70S6K) that is activated in the mTOR signaling pathway. For HCC-derived primary cells from the four HCC patients, the viabilities of TS cells were suppressed by chromeceptin treatment (Fig. 5E). These data suggested that IGF2 plays a role in maintaining stem cell property and TS viability through the pAKT and p-mTOR pathway in Hep3B cells.

### miR-100 and miR-125b mimics affect the inhibition of tumor growth in vivo

**Figure 6:**
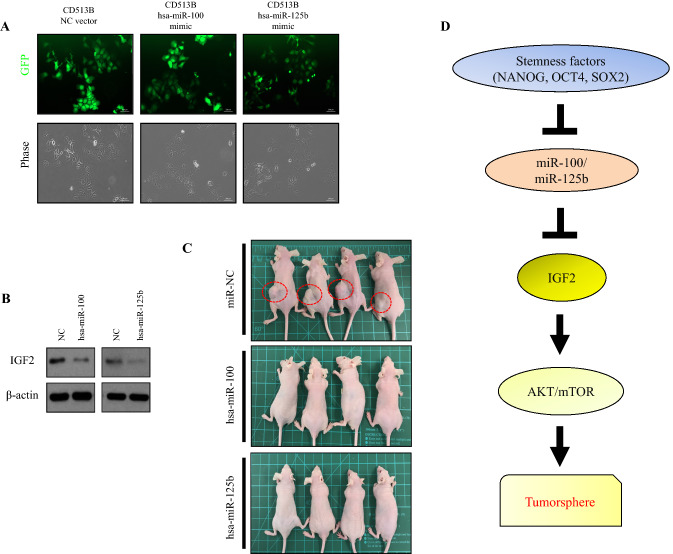
miR-100 and -125b overexpression attenuates liver tumor growth in vivo. (**A**) Fluorescence microscopy examination of Hep3B cells transfected with lentiviral vectors. GFP expression was observed by fluorescence microscopy. Hep3B cells infected with CD513B-control vector or CD513B miR-100b mimic or CD513B miR-125b mimic were detected as green fluorescence in dark field microscopy. Images were taken with an Carl Zeiss Axio Observer.Z1 fluorescence microscope using ZEN software. (**B**) Immunoblots for IGF2 protein in spheres with CD513B-miR-100 or miR-125b mimic. Control vector-transduced cells were used as a control. β-actin was used as an internal control. Blots were cropped to show specific bands. (**C**) Representative images showing the sizes of tumors injected with miR-NC or miR-100, -125b at 30 days. (**D**) Proposed model illustrating the mechanism of miR-100HG in regulating IGF2 and cancer stem cell (CSC) properties in hepatocellular carcinoma (HCC).

To determine the effects of miR-100 and miR-125b on tumorigenicity in vivo, we constructed the stable pCD513B-eGFP vector with hsa-miR-100, hsa-miR-125b mimic, or NC (Fig. 6A). The eGFP-expressing Hep3B cells that also expressed miR-100 and miR-125b were cultured using the TS culturing condition for 7 days. The stable TS cells expressing miR-100 or miR-125b displayed decreased IGF2 protein expression when compared with the NC vector (Fig. 6B). Finally, we injected 3 × 10^5^ cells with forced expression of miR-100, miR-125b, or NC into the flank of male nude mice. The injected TS cells with NC formed palpable tumors by 3 weeks after the injection (Fig. 6C), while xenograft tumors were not seen in mice injected with miR-100 or miR-125b-expressing TS cells. The data suggested potential tumor suppressor roles of miR-100 and miR-125b, possibly through inhibition of IGF2 expression.

## Discussion

CSCs comprise a small portion of the cancer cell population having the capacity of self-renewal. CSCs are critical in tumorigenesis, progression, and metastasis^1,6^. CSCs can be enriched in vitro by culturing in conditions that promote sphere formation with distinct gene expression from 2D adherent cultures^24^. Patient-derived short-term cultured cells from HCC tissues also can be used for sphere forming culture to enrich CSCs^15^. Using PCC of HCC patients, the NanoString miRNA array revealed significant downregulation of miR-100 and miR-125b in TS compared to 2D cells, suggesting possible roles in CSCs. miR-100 and miR-125b are reported to be downregulated and act as tumor suppressors in various cancers, including HCC^16,25,26^. These two miRNAs suppress metastasis of HCC through disruption of angiopoetin2, resulting in inhibition of vessels that encapsulate tumor clusters^25^. Moreover, miR-125b attenuates HCC malignancy by targeting SIRT6^27^. Low expression of miR-100 and miR-125b has been significantly associated with worse prognosis of HCC^28,29^. The miR-125b family members provide a proliferative advantage to cells upon their overexpression, decrease the rate of apoptosis by downregulating proapoptotic genes and promote differentiation toward the myeloid lineage in mice^14,30–33^. Moreover, miR-100 is associated with human mesenchymal stem cell differentiation and the promotion of osteogenesis. Ectopic expression of miR-100 and miR-125b attenuated tumorigenicity of HCC cells through the downregulation of IGF2 (Fig. 6B,C).

miR-100, miR-125b, and let-7a-2 are transcribed from the third intron of MIR-100HG, a polycistronic miRNA host gene located on chromosome 11. However, the expression patterns of let-7a are distinct from the other two miRNAs in various cancers. In pancreatic ductal adenocarcinoma, transforming growth factor-beta induces the transcription of MIR-100HG, resulting in the upregulation of miR-100 and miR-125b, while let-7a is unchanged^22^. MIR-100HG-derived miR-100 and miR-125b have been correlated with cetuximab resistance via Wnt/β-catenin signaling, but not let-7a-2^1^. Our miRNA microarray data also showed a decoupling pattern among the three miRNAs; miR-100 and miR-125b were downregulated in TS cells compared to 2D cells, while no significant change was evident in let-7a expression.

SOX2, OCT4, and NANOG are critical regulators of self-renewal and pluripotency of embryonic stem cells (ESCs), and positively or negatively regulate ESC-specific miRNAs^6^. OCT4/SOX2 directly increases the expression of miR-200 clusters located on chromosome 4 (mir-200a/b/429) and chromosome 6 (mir-141/ 200c) by binding at their promoter regions, and thereby promotes the reprogramming of induced pluripotent stem cells. In contrast, OCT4 binds to the miR-145 promoter, resulting in transcriptional inhibition of miR-145 expression in human ESCs^34^. The promoter region of MIR-100HG has been identified using the luciferase assay, and the binding site of the ETS Like-1 (ELK1) transcription factor located at -1332 to -1323 is responsible for ELK1-induced transcriptional activation^35^. In this study, overexpression of three stemness factors significantly reduced the luciferase activity of the promoter of MIR-100HG. The results suggest that SOX2, OCT4, and NANOG act as transcriptional repressors of MIR-100HG. Since putative OCT4, SOX2, and NANOG binding sites are predicted in the promoter region, the stemness factors may bind to the regions and transcriptionally suppress miR-100 and miR-125b.

IGF2 has oncogenic activity and is overexpressed in diverse types of cancers, including HCC^36^. Overexpression of IGF2 increases sphere forming activity and in vivo tumorigenicity of breast cancer cells^37^. DNA methylation of the IGF2 promoter region is impaired in cancer cells with IGF2 overexpression, suggesting that epigenetic alteration is one of the mechanisms of transcriptional IGF2 activation^38^. In addition, IGF2 expression is reported to be suppressed by miR-100 and miR-125b in breast cancer and C2C12 immortalized mouse myoblast cells^20,39^. We also demonstrated that IGF2 is a downstream common target of miR-100 and miR-125b in TS cells.

When spheroid cultures of HCC PCCs were treated with IGF signaling pathway inhibitor, sphere formation was suppressed. The result suggested that IGF2 is critical to maintaining the formation of TSs. The IGF2-mediated PI3K/AKT/mTOR signaling pathway was activated in HCC TSs, consistent with the previous report that IGF2 activates this pathway^40^. Thus, our data suggest that the downregulation of miR-100 and miR-125b, and the resultant activation of the PI3K/AKT/mTOR pathway through IGF2 overexpression contribute to stemness properties when patient-derived HCC cells were cultured in ultra-low attachment dishes (Fig. 6D). In conclusion, we describe a tumor suppressor role of miR-100 and miR-125b in association with CSCs of HCC that involves the inhibition of IGF2 expression and resultant suppression of the IGF2-mediated PI3K/AKT/mTOR pathway. When these two miRNAs were downregulated by the SOX2, OCT4, and NANOG stemness factors, HCC cells expressed IGF2 and activated the PI3K/AKT/mTOR pathway, resulting in maintenance of CSCs.

## Materials and methods

### Ethics approval and patient-derived PCC culture

This study was conducted in accordance with the Declaration of Helsinki. It was approved by the Human Research Ethics Committee of the Asan Medical Center (IRB-20120112). The institutional review board at the Asan Medical Center complied with related laws such as the International Council for Harmonization of Technical Requirements for Pharmaceuticals for Human Use (ICH) and bioethics and safety acts, and written informed consent was obtained from all patients. The tumor tissues were dissociated and cultivated either on collagen-coated plates for 2D-culture or on low attachment plates with stem cell medium for TS culture according to our previous report^15^.

### NanoString miRNA microarray

Total RNA was extracted from 2D-cultured and spheroid-cultured HCC cells using TRIzol (Life Technologies, Vienna, Austria). To analyze differences in miRNA expression, the nCounter Human miRNA Expression Assay kit (NanoString Technologies) was used according to the manufacturer's instructions.

### Real-time quantitative RT-PCR (qRT-PCR)

RNA samples were reverse transcribed and real-time qRT-PCR analyses were performed. For mature miRNA detection, TaqMan assays were used according to the manufacturer's recommendations (Life Technologies). The assay IDs were RNU6B (#001093), hsa-miR-100-5p (#000437), and hsa-miR-125b-5p (#000449). Expression values were calculated using a comparative Ct method with U6-snRNA (RNU6B) as the endogenous control for qRT-PCR of mRNA, total RNA was reverse transcribed using random primers (Invitrogen, Carlsbad, CA) and the SYBR Green quantitative qPCR (Bio-Rad, Hercules, CA) was performed. The primer sequences used in this study are shown in Supplementary Table  2.

### Reagents

The miRNA mimic, scrambled negative control oligonucleotides, were obtained from Genolution Inc. (Seoul, Korea). For transfection, the reagent was Lipofectamine 2000 (Invitrogen). Chromeceptin was purchased from Sigma-Aldrich (#C0868; St. Louis, MO).

### Immunoblotting

Immunoblot was conducted as previously described^15^ and the following primary antibodies were used in this study: anti-NANOG (#4903; Cell Signaling Technologies, Danvers, MA) anti-SOX2 (#3579; Cell Signaling Technologies), anti-OCT4 (#2840; Cell Signaling Technologies), anti-IGF2 (ab9574; Abcam, Cambridge, UK), phospho-AKT pathway antibody sampler kit (#9916; Cell Signaling Technologies), mTOR pathway antibody sampler kit (#9964; Cell Signaling Technologies), and anti-β-catenin (sc-47778; Santa Cruz Biotechnology, Dallas, TX).

### Dual-luciferase assay

Two regions in the 3′ untranslated region (UTR) of IGF2 harboring the predicted binding sites of miR-100 and miR-125b were amplified from human genomic DNA (Supplementary Table  2) and then cloned into the SacI and XhoI restriction sites of the pmiR GLO control (Promega, Madison, WI). For reporter assays, Hep3B cells were transfected with reporter plasmid and mimic hsa-miR-100 and hsa-miR-125b using Lipofectamine 2000 (Invitrogen). Reporter assays were conducted with the Dual-Luciferase Assay-System (Promega), and the expression levels were normalized by co-transfected Renilla luciferase.

### Stable mimic miR-100 cluster-expressing cells

A recombinant lentivirus containing a Puro-GFP-CD513B vector was obtained from System Biosciences (Palo Alto, CA). Virus production and transduction were performed following the manufacturer's protocol. The resulting pmiR-Ctrl and pmiR-100 cluster cells that stably overexpressed the miR-100 cluster were selected using puromycin (1 µg/ml). These lentiviruses were used to infect Hep3B cells, and fluorescence-activated cell sorting was used to obtain cells that stably expressed enhanced green fluorescence protein (eGFP)-miR-100 and eGFP-miR-125b.

### Cell viability

Tumorsphere (TS) viability was estimated using the Cell Titer-Glo Luminescent Cell Viability Assay Kit (Promega, Wisconsin, US). Luminescence was measured using Victor 3 1420 Multilabel Counter (Perkin Elmer, Villebon-sur-Yvette, France).

### In vivo tumorigenicity

All experiments were accordance with national guidelines and regulations, and with the approval of the animal care and use committees at ASAN medical center. The animal protocol was conducted strictly in accordance to the national institute of health guide for the care and use of laboratory animals. To assess the effects of miR-100 and miR-125b expression on tumorigenicity in vivo, the lentivirus infected Hep3B cells were used for TS culture. The cells were xenografted into nude mice, using 3 × 10^5^ cancer cells in 100 µl Growth Factor Reduced Matrigel (Cat #354230, Lot #3010866; BD Bioscience, Santa Clara, CA) per mouse.

### Statistical analysis

All experiments were repeated at least three times. The values in the figures are presented as means ± standard deviation (SD). Animal studies included seven animals per group. The statistical analysis involved two-way analysis of variance (ANOVA) in which *p* < 0.05 was considered as significant.

## Supplementary information

Supplementary information.
