# Supplementary material for: Loss of miR-100 and miR-125b results in cancer stem cell properties through IGF2 upregulation in hepatocellular carcinoma
Source: Sci Rep. 2020 Dec 8;10:21412. doi: 10.1038/s41598-020-77960-9 (PMC7722933; doi:10.1038/s41598-020-77960-9)
Supplement: Supplementary file 1 — Supplementary information. [file 41598_2020_77960_MOESM1_ESM.pdf]

Supplementary Information

Loss of miR-100 and miR-125b results in cancer stem cell properties through IGF2 upregulation in hepatocellular carcinoma

Hyang Sook Seol, Yoshimitsu Akiyama, San-Eun Lee, Shu Shimada, Se Jin Jang.

1. Supplementary Figure 1
Tumorspheres and two-dimensionally culturing cells

2. Supplementary Figure 2
Stemness factor expression is increased with time during culture

3. Supplementary Figure 3
Expression of miR-100 and miR-125b after transfection of such miRNA mimic

4. Supplementary Table 1
Representative differentially expressed genes between tumorsphere (TS) and adherent cells (2D)

5. Supplementary Table 2
List of primer sequences used in this study

6. Supplementary Figure 4
Uncropped scans of Immunoblots

Supplementary Figure 1. Tumorshperes and two-dimensionally culturing cells

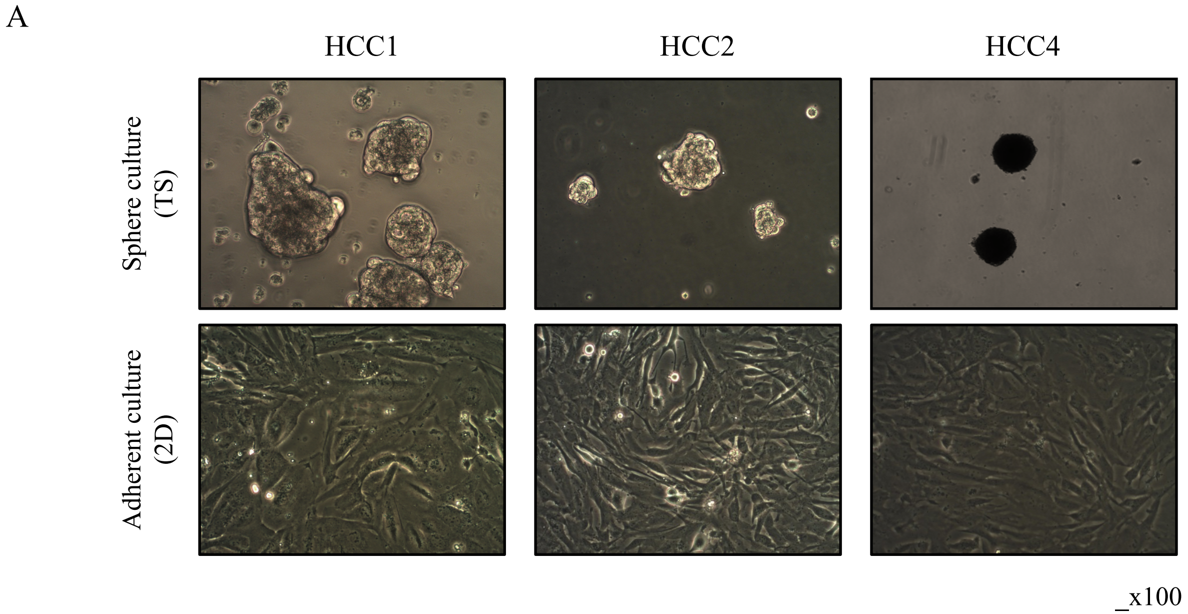

(A) Hepatocellular primary cancer cells derived from patients were cultured in ultra-low attachment dishes with CSC culture medium as described in the Materials and Method for 7 days to enrich CSCs. The spheres formed under the CSC culture condition, as well as monolayer cells cultured in collagen type 1 coated dishes.

Supplementary Figure 2. Stemness factor expression is increased with time during culture

A

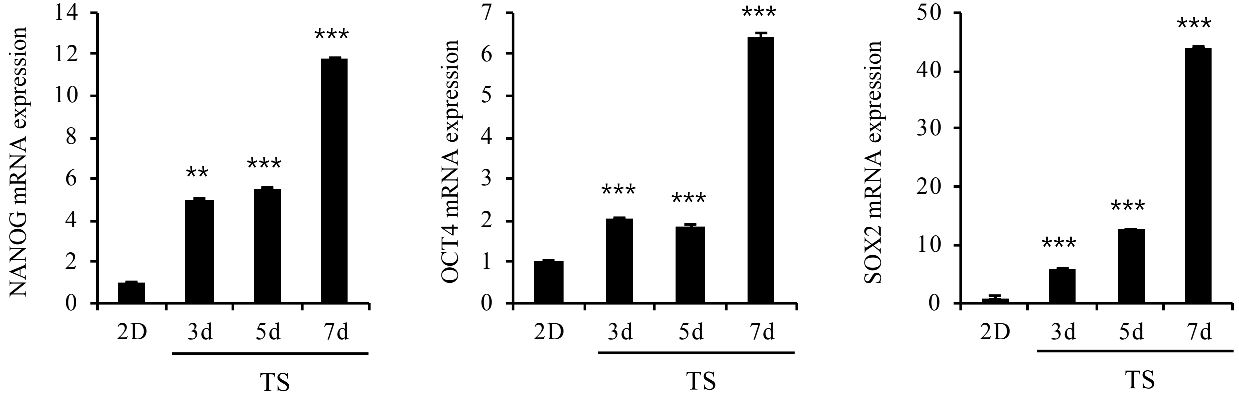

(A) Hepatocellular carcinoma cells (Hep3B) was cultured in ultra-low attachment dishes with CSC culture medium. After harvesting following 3, 5, and 7 days of culture, stemness factor mRNA expression was confirmed with qRT-PCR ($^{**}p$<0.01, $^{***}p$<0.001).

Seol et al.,

Supplementary Figure 3. Expression of miR-100 and miR-125b after transfection of such miRNA mimic

A

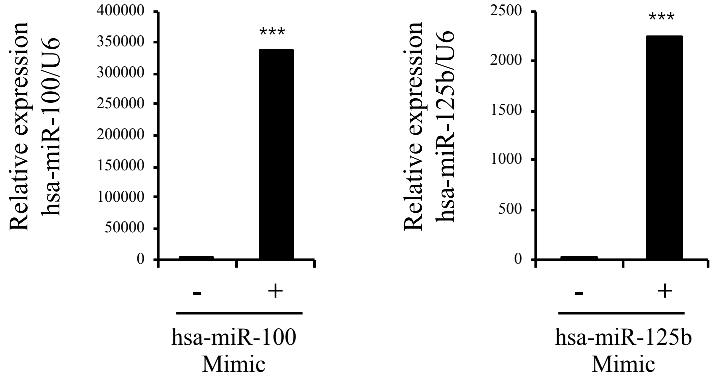

(A) qRT-PCR analysis of miR-100 and miR-125b expression in scramble or miR-100HG mimic transfected in Hep3B. Relative expression was calculated using U6 expression as an internal control. The average (column) ± SD (bar) is indicated (***p<0.001).

Supplementary Table 1. Representative differentially expressed genes between tumorsphere (TS) and adherent cells (2D)

Gene	2D vs TS Up-regulated genes			Gene	2D vs TS Down-regulated genes		
	Accession	FC	*p*-value		Accession	FC	*p*-value
CXCL12	NM_000609.4	3.495399	1.19751E-11	CXCL5	NM_002994.3	-10.458881	8.80225E-61
ANKRD37	NM_181726.2	3.460855	7.80283E-13	IL8	NM_000584.2	-9.759449	1.49158E-65
AGR2	NM_006408.2	3.441012	1.72259E-13	ID1	NM_181353.1	-7.997096	2.88753E-67
KCNIP4	NM_001035004.1	3.284018	2.06944E-11	LOXL4	NM_032211.6	-6.676209	2.07606E-45
RDH12	NM_152443.1	3.230006	2.18329E-10	IGFBP7	NM_001553.1	-6.519419	2.50048E-42
DACH1	NM_080760.3	3.226897	6.33637E-13	FBN2	NM_001999.3	-5.742478	1.60064E-34
SLC38A4	NM_018018.2	3.212169	4.10459E-10	KRT23	NM_015515.3	-5.679952	1.07045E-44
CEACAM1	NM_001712.3	2.952201	9.26112E-10	SGK1	NM_005627.3	-5.602522	5.52859E-35
PGF	NM_002632.4	2.943501	9.72913E-08	CXCL1	NM_001511.1	-5.450926	1.93092E-29
PRKD1	NM_002742.2	2.937324	6.46297E-11	CUX2	NM_015267.2	-4.884542	2.88348E-21
SERPINI1	NM_005025.2	2.756771	6.00447E-14	ANXA3	NM_005139.2	-4.793872	3.91825E-30
FAM131C	NM_182623.1	2.752774	5.50219E-11	FGB	NM_005141.2	-4.518927	1.68543E-32
**IGF2**	NM_001007139.3	2.482884	3.04324E-06	LEPREL1	NM_018192.2	-4.432207	5.48236E-20
FGL1	NM_201552.1	2.482590	1.12067E-06	CTGF	NM_001901.2	-4.300840	1.28565E-29
MAF	NM_005360.3	2.482058	4.55423E-07	CHST9	NM_031422.2	-4.166967	1.9615E-23
UGT2B4	NM_021139.1	2.479903	3.03189E-06	SCN9A	NM_002977.1	-4.133820	1.38401E-20
AQP12A	NM_198998.1	2.477471	4.63673E-05	SAMD11	NM_152486.2	-4.103351	1.05953E-15
KLF6	NM_001008490.1	2.470973	8.30228E-07	CXCL5	NM_002994.3	-4.073478	1.70654E-18
HIST2H2AA4	NM_001040874.1	2.468602	6.3627E-08	GJA1	NM_000165.3	-4.068281	4.81821E-24
HPN	NM_002151.1	2.467558	2.50891E-05	ANKRD38	NM_181712.3	-3.919848	1.16737E-17

Abbreviations: TS, tumor sphere; 2D, adherent cells; FC, fold-change

Supplementary Table 2. List of primer sequences used in this study

	Gene	Sense (5' to 3')	Antisense (5' to 3')
qRT-PCR	NANOG	TTC CTT CCT CCA TGG ATC TG	TCT GCT GGA GGC TGA GGT AT
	Oct4A	GAA GGA TGT GGT CCG AGT GT	GTG AAG TGA GGG CTC CCA TA
	SOX2	AAC CCC AAG ATG CAC AAC TC	CGG GGC CGG TAT TTA TAA TC
	IGF2	CAT CGT TGA GGA GTG CTG TTT	GCT TCC AGG TGT CAT ATT GGA
	18s	GTA ACC CGT TGA ACC CCAT T	CCA TCC AAT CGG TAG TAG CG
Reporter assay	IGF2_1	GGG AGC TCG CTG AGA AAC AAT TGG CAA AA	GGC TCG AGT GCG GGG ATG CAT AAA GTA T
	IGF2_2	GGG AGC TCG GGG CTA GCA GGT GTG TAA A	GGC TCG AGA TTT GGC TCT TTG GGC TTC T

Two regions in the 3′ UTR of IGF2 were amplified with the primer sets (1; -458 to -878, 2; -2,817 to -3,375, Genbank accession No.NM_000612) containing the SacI (GAGCTC) or XhoI (CTCGAG) sequences (Figure 4D).

Supplementary Figure 3. Uncropped scans of Immunoblots

Figure 3B

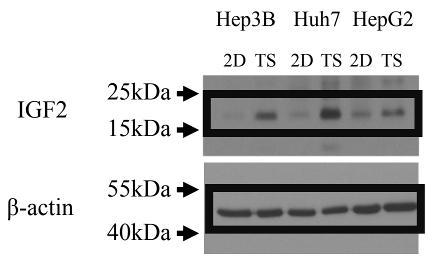

Figure 3E

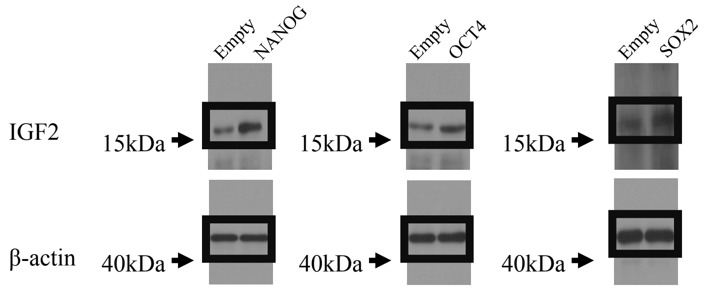

Figure 4B

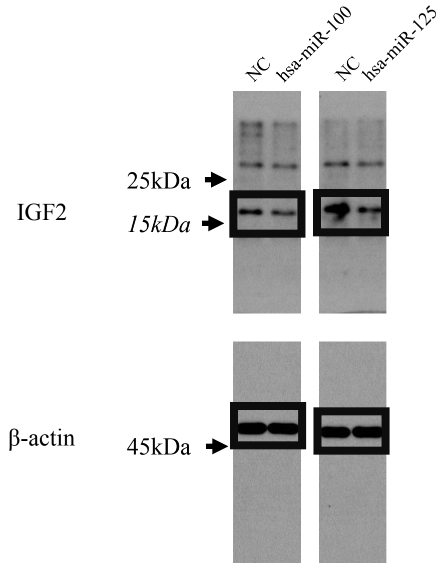

Figure 4C

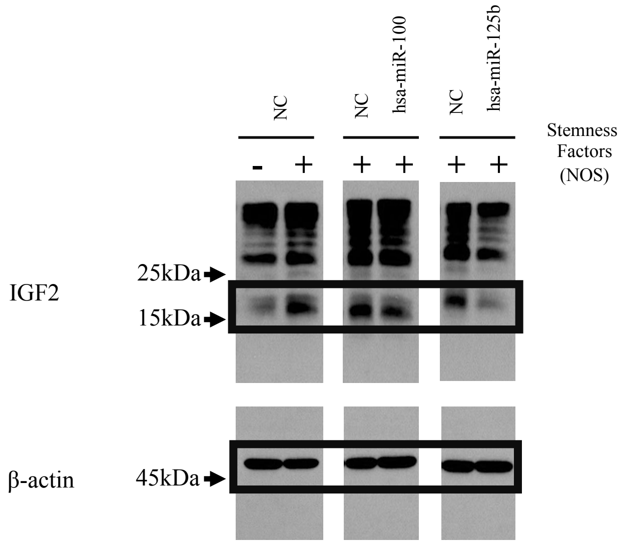

Figure 5A

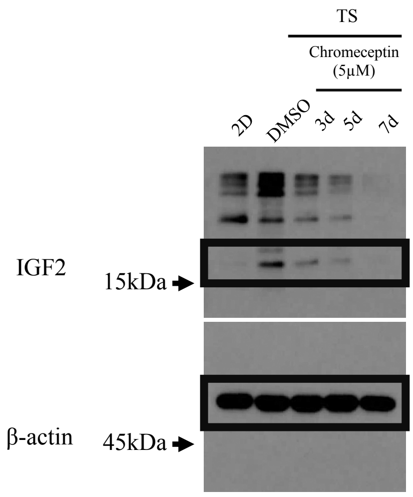

Figure 6B

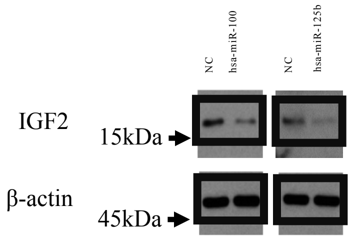

Figure 5D

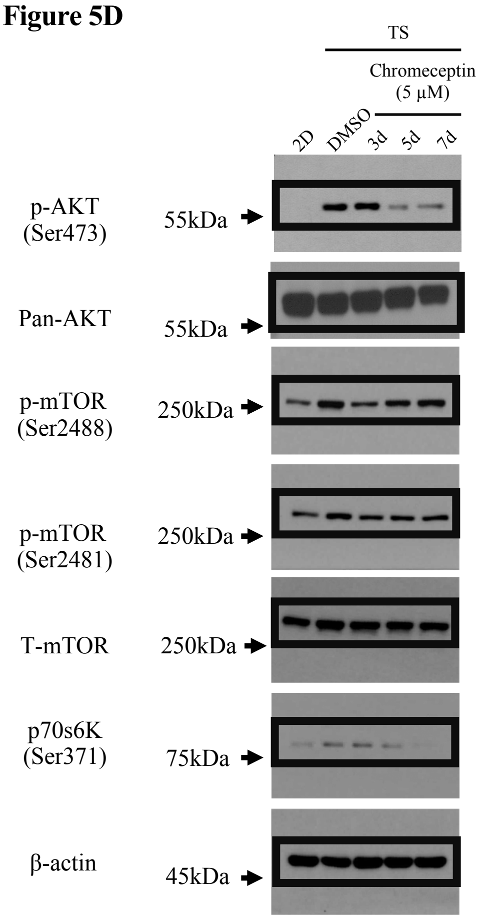

Seol et al.,
